# Influenza H5N1 and H1N1 Virus Replication and Innate Immune Responses in Bronchial Epithelial Cells Are Influenced by the State of Differentiation

**DOI:** 10.1371/journal.pone.0008713

**Published:** 2010-01-15

**Authors:** Renee W. Y. Chan, Kit M. Yuen, Wendy C. L. Yu, Carol C. C. Ho, John M. Nicholls, J. S. Malik Peiris, Michael C. W. Chan

**Affiliations:** 1 Department of Microbiology, Li Ka Shing Faculty of Medicine, The University of Hong Kong, Queen Mary Hospital, Pokfulam, Hong Kong SAR, People's Republic of China; 2 Department of Pathology, Li Ka Shing Faculty of Medicine, The University of Hong Kong, Queen Mary Hospital, Pokfulam, Hong Kong SAR, People's Republic of China; 3 HKU-Pasteur Research Centre, Pokfulam, Hong Kong SAR, People's Republic of China; Charité-Universitätsmedizin Berlin, Germany

## Abstract

Influenza H5N1 virus continues to be enzootic in poultry and transmits zoonotically to humans. Although a swine-origin H1N1 virus has emerged to become pandemic, its virulence for humans remains modest in comparison to that seen in zoonotic H5N1 disease. As human respiratory epithelium is the primary target cells for influenza viruses, elucidating the viral tropism and host innate immune responses of influenza H5N1 virus in human bronchial epithelium may help to understand the pathogenesis. Here we established primary culture of undifferentiated and well differentiated normal human bronchial epithelial (NHBE) cells and infected with highly pathogenic influenza H5N1 virus (A/Vietnam/3046/2004) and a seasonal influenza H1N1 virus (A/Hong Kong/54/1998), the viral replication kinetics and cytokine and chemokine responses were compared by qPCR and ELISA. We found that the *in vitro* culture of the well differentiated NHBE cells acquired the physiological properties of normal human bronchi tissue which express high level of α2-6-linked sialic acid receptors and human airway trypsin-like (HAT) protease, in contrast to the low expression in the non-differentiated NHBE cells. When compared to H1N1 virus, the H5N1 virus replicated more efficiently and induced a stronger type I interferon response in the undifferentiated NHBE cells. In contrast, in well differentiated cultures, H5N1 virus replication was less efficient and elicited a lower interferon-beta response in comparison with H1N1 virus. Our data suggest that the differentiation of bronchial epithelial cells has a major influence in cells' permissiveness to human H1N1 and avian H5N1 viruses and the host innate immune responses. The reduced virus replication efficiency partially accounts for the lower interferon-beta responses in influenza H5N1 virus infected well differentiated NHBE cells. Since influenza infection in the bronchial epithelium will lead to tissue damage and associate with the epithelium regeneration, the data generated from the undifferentiated NHBE cultures may also be relevant to disease pathogenesis.

## Introduction

Highly pathogenic avian influenza (HPAI) H5N1 virus continues to be enzootic in poultry in parts of Asia and Africa and transmits zoonotically to humans. From 2003 to November 2009, influenza H5N1 virus has caused 444 confirmed human cases and 262 of them were fatal. Human H5N1 cases were found in 15 countries; the three most affected countries being Vietnam, China and Indonesia, where the fatality rates ranged from 42–82% [1]. Although a swine origin influenza H1N1 virus (H1N1pdm) has recently emerged to become pandemic, its virulence for humans so far remains modest in comparison with that seen in zoonotic H5N1 disease [2]. As H5N1 virus continues to pose a threat to human health zoonotically and may still become more efficiently transmissible in humans through reassortment with the novel pandemic H1N1 virus or other means, it is important to understand the determinants of virus replication and its' pathogenesis in humans. Furthermore, elucidating the pathogenesis underlying the unusual virulence of H5N1 virus may help understand the pathogenesis of acute respiratory distress syndrome in severe viral pneumonia, including that seen occasionally in pandemic H1N1 [3].

An understanding of the pathogenesis of H5N1 infection in humans may derive from the study of human disease, relevant animal models, and primary human cells infected with virus *in vitro* or *ex vivo* [4]–[11]. A comparison of H5N1 virus with seasonal influenza viruses (H1N1 and H3N2) is likely to provide insights into the unusual severity of H5N1 disease in humans. Previously we used undifferentiated primary normal human bronchial epithelial (NHBE) cells and alveolar epithelial cells to evaluate the virus replication kinetics and the innate immune responses induced by H5N1 virus compared with the seasonal H1N1 virus. Undifferentiated NHBE cells were readily infected with both seasonal H1N1 and avian H5N1 viruses. But H5N1 influenza virus induced exceptionally high levels of cytokines and chemokines when compared to the contemporary human influenza H1N1 virus [5]. On the other hand, a recent publication by Zeng *et al*. [12] indicated that the highly pathogenic influenza H5N1 viruses elicits an attenuated type I interferon (IFN) response in the polarized bronchial epithelial cell line (Calu-3) when compared to a human influenza H3N2 virus. This apparent discrepancy in the reported literature prompted us to compare viral replication and host responses in undifferentiated (ud) and well differentiated (wd) NHBE cells in parallel. We hypothesized that the “attenuation” of host innate immune response by influenza H5N1 virus infection in wd-NHBE cells model may be due to the relative restriction of viral replication efficiency in wd-NHBE cells. We also aimed to compare the profile of the sialic acid (Sia) receptors that bind influenza viruses and the physiological properties of these two *in vitro* cell culture models with that seen in human bronchial tissue by using lectin histochemistry, a standard method for detection of the Sia linkages [13]. The influenza virus replication kinetics and the cytokine and chemokine responses in these cells infected with influenza A viruses, A/Hong Kong/54/98 (H1N1) and A/Vietnam/3046/04 (H5N1) were compared. In summary, we demonstrated that the level of differentiation of NHBE has a profound effect on the expression of Sia receptors, virus replication competence and cytokine responses, including the type I interferon. Furthermore, H5N1 and H1N1 viruses behaved differently in the ud- and wd-NHBE cells. These results emphasized the importance of using a physiologically relevant respiratory epithelium model to study the pathogenesis of influenza virus.

## Materials and Methods

### Viruses

A highly pathogenic influenza H5N1 virus, A/Vietnam/3046/2004 (hereafter referred to as VN04/H5N1), a clade 1 H5N1 virus isolated from a patient with fatal influenza H5N1 disease in Vietnam in 2004 and a seasonal human influenza H1N1 virus, A/Hong Kong/54/98 (hereafter referred to as HK98/H1N1), a representative seasonal influenza virus isolated in Hong Kong in 1998, were used for our comparative studies. The viruses were initially isolated and passaged in Madin-Darby canine kidney (MDCK) cells. The virus stock was aliquoted, and titrated to determine tissue culture infection dose 50% (TCID_50_) in MDCK cells as described in our previous study [4]–[6], [11]. The experiments were carried out in a Bio-safety level 3 (BSL-3) facility at the Department of Microbiology, The University of Hong Kong.

### Normal Human Bronchial Epithelial (NHBE) Cells

NHBE cells (Lonza, Walkersville, Inc.) were purchased as cryopreserved vials. NHBE cells at passage 3 to 5 were used in this study.

### Culture of ud-NHBE Cell

NHBE cells were grown and subcultured according to the suppliers instructions in serum-free and hormone supplemented bronchial epithelial growth media (BEGM) (Lonza, Walkersville, Inc.) as described [5]. The cell suspensions were seeded on transwell inserts (Corning, New York, USA) with a cell density of 1×10^5^ cells/cm^2^ and cells were incubated in a humidified atmosphere (5% CO_2_, 37°C) under submerged conditions.

### Culture of wd-NHBE Cell

NHBE cells were plated into a T175 tissue culture grade culture flask in a density of 500 cells/cm^2^ for cell proliferation. Bronchial epithelial basal medium (BEBM) (Lonza Walkersville, Inc.) was supplemented with growth factor and hormones as previously described [14]. After subculture, they were plated to a cell density of 2.5×10^5^ cells/cm^2^ on human collagen IV (BD Science) coated transwell inserts (Corning) [15]. BEBM culture medium was supplemented as previously described [16]–[18] with the retinoic acid concentration adjusted to 10^−7^ M. Medium was changed every 48 h until the cell layer reached confluence. Air liquid interface (ALI) culturing environment was then established by removing the culture medium from the apical compartment. Thereafter, medium was changed in the basolateral compartment every 48 h until day 21 of ALI culture. The apical compartment was gently washed with phosphate buffered saline (PBS) once a week to remove accumulated mucus and debris [16]. The transepithelial resistance was measured by EVOM epithelial voltohmeter (World Precision Instruments, Sarasota, Fla.). At day 21 of ALI culture, the NHBE cells became well differentiated and ready for use.

### wd-NHBE Cell Characterization

Immunofluorescence and immunohistochemistry staining was done on ud-NHBE and wd-NHBE cells. The transwell cultures were collected and fixed with 10% formalin, paraffin embedded, and then cross-sectioned for histological examination. Slides were stained using hematoxylin-eosin. Ciliated cells were further identified by FITC-conjugated β-tubulin antibody (Sigma, Saint Louis, USA) and goblet cells were identified by biotinylated MUC5AC antibody (Invitrogen, San Francisco, USA). Moreover, the expression of human airway trypsin-like (HAT) protease was detected by RT-qPCR using the mRNA extracted from the NHBE cultures. Human bronchial tissue was also stained with β-tubulin and MUC5AC. Tissues were obtained from lobectomy or pneumonectomy specimens of patients having surgical resection of lung tissue. The study was approved by The Hong Kong University and Hospital Authority (Hong Kong West) Institutional Review Board.

### Lectin Histochemistry

The lectin *Sambucus nigra* agglutinin (SNA) used primarily detects Sia-α2-6-linkages and *Maackia amurensis* lectin (MAL)-I and MAL-II which identifies two Sia-α2-3Gal linkages: Sia-α2-3Galβ1-4GlcNAc and Sia-α2-3Galβ1-3GalNAc respectively. The antigen retrieval and staining of the paraffin sections of the NHBE cells and human bronchial tissues were performed as described [4], [8]. Briefly, the sections were microwaved in citrate buffer and blocked with H_2_O_2_ in Tris buffered saline and with avidin/biotin blocking kit (Vector, Burlingame). They were then incubated with horseradish peroxidase conjugated SNA (EY Laboratories), biotinylated MAL-I (Vector, Burlingame) and 1∶100 biotinylated MAL-II (Vector, Burlingame) and blocked with 1% bovine serum albumin and then incubated with strep-ABC complex (Dako Cytomation, Cambridgeshire, UK). Development was performed using the AEC substrate kit (Vector, Burlingame), the nuclei were counterstained with Mayer's hematoxylin and then the sections were dried and mounted with DAKO aqueous mount (Dako Cytomation, Cambridgeshire, UK).

### Influenza Virus Infection of ud- and wd-NHBE Cells

The ud- and wd-NHBE cell cultures were infected with influenza A viruses at a multiplicity of infection (MOI) of 2 for the analysis of virus replication and at a MOIs of 2 and 5 for the analysis of cytokine and chemokine. MEM medium (Gibco) with 100 U/ml penicillin and 100 µg/ml streptomycin was used as inoculum in the mock infected cells. The cell culture was incubated with the virus inoculum for 1 h in a water-jacketed 37°C incubator with 5% CO_2_ supply. Virus inoculum was discarded after the incubation and the cell culture was rinsed 3 times with warm PBS. The infected cell culture was then replenished by the appropriate growth medium (corresponding to the differentiation state of the NHBE cells).

### Quantification of Influenza Virus Replication

The mRNA of the infected cells were collected at 3 h, 6 h and 24 h post infection for viral gene quantification using qPCR as described [5] and the culture supernatants of infected cells were collected at 1 h, 24 h and 48 h post infection. Productive viral replication were determined by titrating the supernatants from infected cell cultures at 1 h, 24 h and 48 h post infection in quadruplicate in MDCK cells by TCID_50_ assay. The cell layer was also fixed at 16 h post infection using 4% paraformaldehyde for immunofluorescence staining with mouse anti-influenza nucleoprotein and matrix antibody conjugated with FITC (DAKO Diagnostics, IMAGEN Influenza, Dakocytomation, Demark).

### Viral Titration by TCID_50_ Assay

A confluent 96-well tissue culture plate of MDCK cells was prepared one day before the virus titration (TCID_50_) assay. Cells were washed once with PBS and replenished with serum-free MEM medium supplemented with 100 units/ml penicillin and 100 µg/ml streptomycin and 2 µg/ml of TPCK (tosylsulfonyl phenylalanylchloromethyl ketone) treated trypsin. Serial dilutions of virus supernatant, from 0.5 log to 7 log, were performed before adding the virus dilutions onto the plates in quadruplicate. The plates were observed for cytopathic effect daily. The end-point of viral dilution leading to CPE in 50% of inoculated wells was estimated using the Karber method [19].

### Quantification of Cytokine and Chemokine by RT-qPCR and ELISA

mRNA from infected cells was extracted at 1 h, 3 h, 6 h and 24 h post infection using RNeasy Mini kit (Qiagen, Hilden, Germany) and treated with DNase. cDNA was synthesized with Oligo-dT primers and Superscript III reverse transcriptase (Invitrogen) and quantified by real-time quantitative PCR analysis with a LightCycler (Roche, Mannheim, Germany). The gene expression profile for cytokines, TNF-alpha (TNF-α) and interferon beta (IFN-β) and chemokines (IP-10, RANTES) and viral matrix gene were quantified and normalized using the housekeeping gene product β-actin mRNA. The primers and methods used for these assays have been reported previously [5], [6]. The concentrations of IFN-β, RANTES and IP-10 proteins in culture supernatants collected at 8 h or 24 h post infection of the influenza viruses infected NHBE cells were measured by ELISA assay as recommended by the manufacturer (R&D Systems, Minneapolis, MN, USA).

### Statistical Analysis

Two-tailed student *t*-test was used to compare the differences of viral titers at different time points post-infection and between different viruses. The quantitative cytokine and chemokine mRNA and protein expression profile of mock, influenza H1N1 and H5N1 virus infected cells was compared using one-way ANOVA, followed by Bonferroni multiple-comparison test. Differences were considered significant at *p*<0.05. The statistical analysis was carried out using Graph-pad Prism 5.

## Results

### Bronchial Epithelial Cells Differentiation

**Figure 1:**
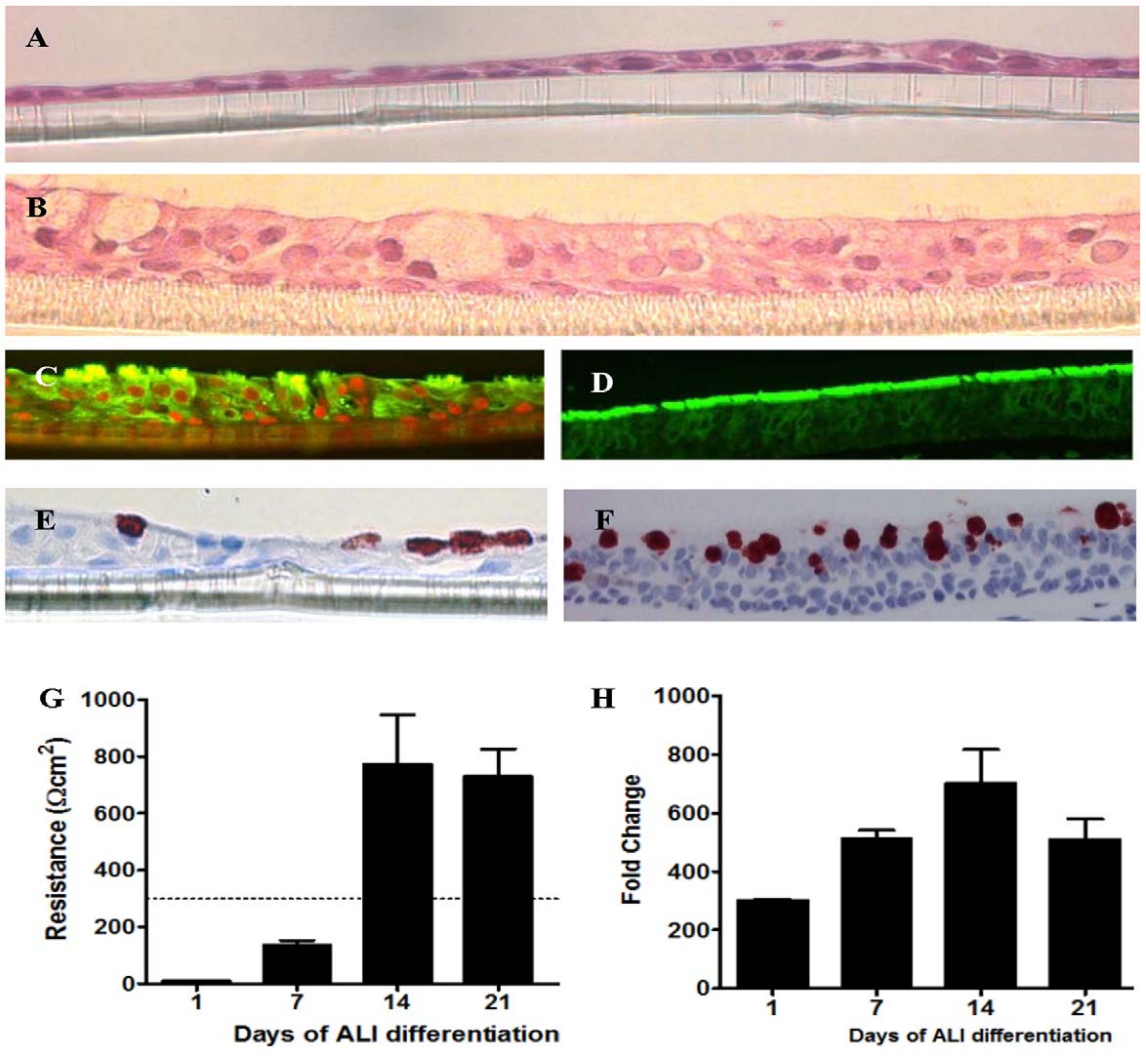
Wd-NHBE cells *in vitro* cultured in ALI (A) for 7 days and for (B) 21 days show the pseudostratified columnar type epithelium by H&E staining. (C) Positive staining of FITC-conjugated β-tubulin on apical surface of epithelium confirms the presence of cilia and (E) positive staining of biotinylated MUC5AC indicates the presence of mucin secreting goblet cells. Human bronchus stained with (D) FITC-conjugated β-tubulin and (F) biotinylated MUC5AC showed the presence of both ciliated and mucus secreting goblet cells along the epithelium. (G) Transepithelial resistance and (H) HAT mRNA expression by of the differentiating NHBE culture from D1 to D21 ALI culture.

The hematoxylin and eosin staining of the NHBE cells after 7-days **(** **Figure 1A** **)** and 21-days (Figure 1B) of ALI culture. For the cultures of the wd-NHBE cells with 21-days in ALI culture, it showed pseudostratified columnar epithelium structure with ciliary projections **(** **Figure 1B** **)**. The ciliated cells on the wd-NHBE cells stained positive with β-tubulin **(** **Figure 1C** **)** and goblet cells were stained positive with MUC5AC **(** **Figure 1E** **)**. A similar staining pattern of ciliated **(** **Figure 1D** **)** and goblet cells **(** **Figure 1F** **)** was observed in human bronchus. However, the ud-NHBE cells (monolayer cultures and the NHBE cells cultured under 7-days of ALI culture) stained negative with these two cell differentiation markers (data not shown).

We evaluated the transepithelial resistance developed between the apical and basolateral compartment of the NHBE cells in transwell inserts from day 1 to 21 of ALI culture from six independent NHBE cell cultures. The transepithelial resistance started to increase at day 7 and reaches the reference resistance of at least 800 Ω cm^2^ [20] upon day 21 **(** **Figure 1G** **)** which is an indication of tight-junction formation in respiratory epithelium. We also evaluated the mRNA expression level of HAT proteases in wd-NHBE cell cultures and found a 700-fold increase in gene expression in ALI-NHBE cell cultures differentiated for 21 days when compared to the ud-NHBE cells **(** **Figure 1H** **)**.

### Sialic Acid Receptor Distribution on ud- and wd-NHBE Cells

**Figure 2:**
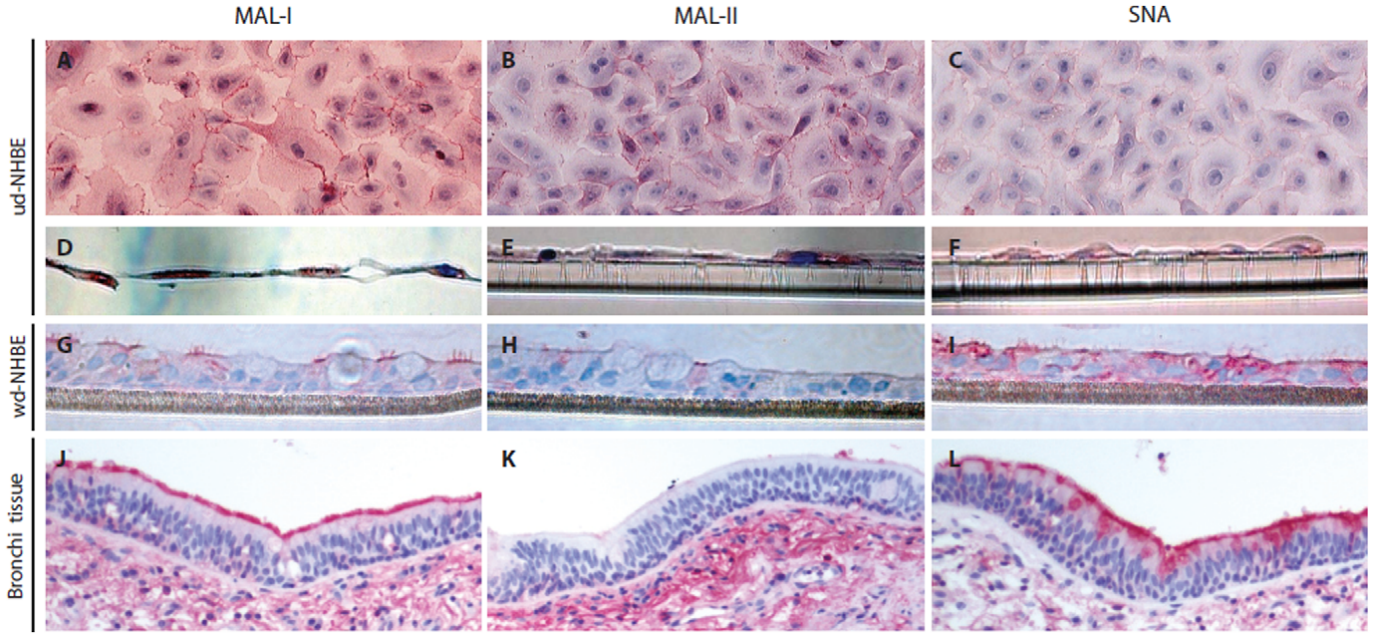
Lectin binding: MAL-I binds to Sia-α2-3Galβ1-4GlcNAc (Panel A, D, G and J), MAL-II binds to Sia-α2-3Galβ1-3GalNAc (Panel B, E, H and K) and SNA binds to Sia-α2-6-linkage (Panel C, F, I and L) to determine the Sias distribution on the ud- and wd-NHBE cells. MAL-I, MAL-II and SNA bindings presented on the (A-C) *en face* staining of ud-NHBE, (D-F) cross-section staining of ud-NHBE, (G-I) cross-section staining of wd-NHBE cells *in vitro* cultures and (J-L) the human bronchial biopsy in reddish brown.

Lectin histochemistry on the primary cultures of human ud- and wd-NHBE cells using SNA (which recognizes the human influenza receptor with Sia-α2-6 linkages) and MAL-I and MAL-II (which recognizes the avian influenza receptor with linkages Sia-α2-3Galβ1-4GlcNAc and Sia-α2-3Galβ1-3GalNAc, respectively). We found that both MAL-I **(** **Figure 2A** **, en face and** **Figure 2D** **, cross-section)** and MAL-II **(** **Figure 2B** **, en face and** **Figure 2E** **, cross- section)** bound strongly and the SNA **(** **Figure 2C** **. en face and** **Figure 2F** **, cross-section)** bound weakly to the ud-NHBE cells indicating that both Sia-α2-3Galβ1-3GalNAc and Sia-α2-6 linkages were present but with different abundance. Thus ud-NHBE cells expressed more avian influenza Sia-α2-3 linkage receptors. On the other hand, the wd-NHBE cells which had acquired the physiological properties of normal human bronchi tissue exhibited strong binding with MAL-I **(** **Figure 2G and 2J** **)** and SNA **(** **Figure 2I and 2L** **)** while MAL-II binding was sparse, focal and confined to ciliated cells **(** **Figure 2H and 2K** **)**. This indicated that Sia-α2-3Galβ1-4GlcNAc and Sia-α2-6 linkages were abundant while Sia-α2-3Galβ1-3GalNAc was comparatively scarce in wd-NHBE cells and human bronchi tissue.

### Replication of Influenza Virus in ud and wd NHBE Cells

**Figure 3:**
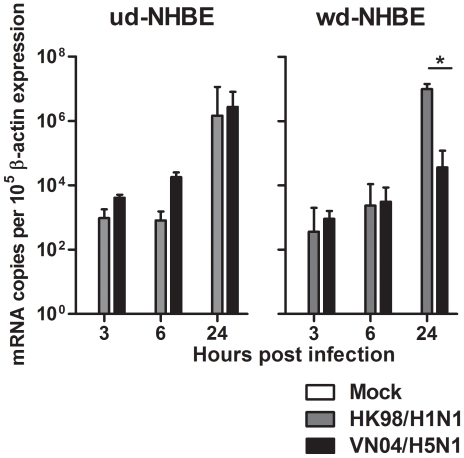
Influenza matrix (M) gene expression after infection of influenza HK98/H1N1 virus (grey bars) and influenza VN04/H5N1 virus (black bars). Ud-NHBE supported M gene transcription from 3 h to 24 h post infection for both influenza viruses while wd-NHBE supported a better influenza HK98/H1N1 virus M gene transcription than influenza H5N1 virus. Bars represented the mean M gene expressed per 10^5^ β–actin house keeping gene and error bar represent the standard error of mean from three independent experiments. Asterisk indicates significant difference with *p*<0.05.

Both influenza A viruses, HK98/H1N1 and VN04/H5N1 were able to infect the ud- and wd-NHBE cells. The influenza matrix (M) gene copy number was measured by quantitative PCR as a measure of viral replication after the infection of both influenza viruses. In ud-NHBE cells, the M gene transcription for both the influenza H1N1 and H5N1 viruses increased comparably from 3 h to 24 h post infection. Similar infection experiments in wd-NHBE cells showed comparable M gene transcription for the two viruses at 3 h and 6 h post infection but there was significantly more M gene mRNA copies found in HK98/H1N1 compared with VN04/H5N1 virus infected wd-NHBE cells by 24 h post infection (*p* = 0.03) **(** **Figure 3** **)**.

**Figure 4:**
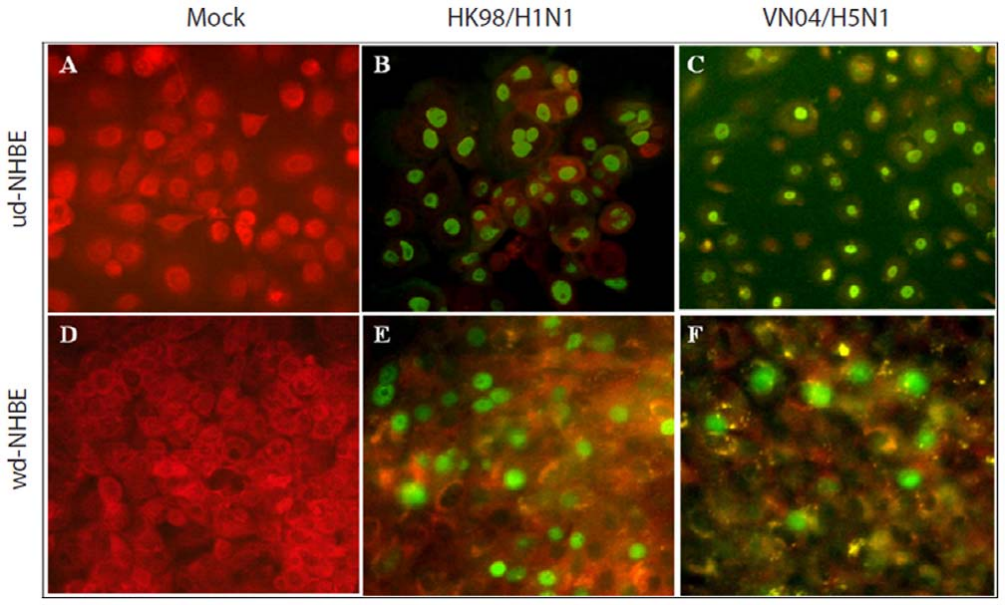
Immunofluorescence staining of (A–C) ud-NHBE cells and (D–F) wd-NHBE cells at 16 h post infection with (A and D) mock infection, (B and E) HK98/H1N1 and (C and F) VN04/H5N1. Influenza nucleoprotein and matrix protein was stained in green with FITC-conjugated mouse antibody.

Influenza viral protein expression was detected by immunofluoresent staining of HK98/H1N1 and VN04/H5N1 virus infected ud- and wd-NHBE cells at 16 h post infection. The ud-NHBE cells were equally susceptible to HK98/H1N1 and VN04/H5N1 virus infection with infection rates of 95.44±4.55% in HK98/H1N1 and 81±4.17% in VN04/H5N1 infected cells **(** **Figure 4B and 4C** **)**. However, in wd-NHBE cell cultures, the percentage of cells infected by influenza VN04/H5N1 virus (8.63±0.69%) was significantly lower (*p* = 0.01) than that by influenza HK98/H1N1 virus (36±4.89%) **(** **Figure 4E and 4F** **).**

**Figure 5:**
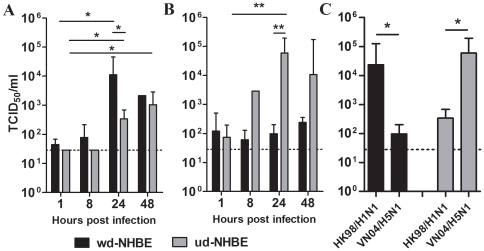
Virus titer detected in the supernatant of influenza virus infected ud- and wd-NHBE cells. Virus titer of the (A) HK98/H1N1 and (B) VN04/H5N1 was determined after influenza viruses infected in the ud- and wd-NHBE cells from 1 h to 48 h post infection at MOI of 2. (C) The comparison of viral replication kinetic between influenza HK98/H1N1 and VN04/H5N1 viruses in ud- and wd-NHBE at 24 h post infection. The chart showed the mean and the standard error of the virus titer pooled from three independent experiments. Single asterisk indicated statistically significant difference of means with *p*<0.05, double asterisks indicated statistically significant differences of means with *p*<0.01. Dotted line represents the detection limit of the TCID_50_ assay.

Infectious virus yield (TCID_50_) assay was quantitated in the supernatants of HK98/H1N1 and VN04/H5N1 infected ud- and wd-NHBE cell cultures to compare infectious virus yields and the replication kinetics. There was evidence of productive viral replication in HK98/H1N1 infected ud-NHBE and wd-NHBE cells **(** **Figure 5A** **)**. The virus titers peaked higher (*p* = 0.04) and earlier in wd-NHBE compared to ud-NHBE cell cultures. VN04/H5N1 titers in ud-NHBE cells peaked at 24 h post infection with a significant increase in the virus titer (*p* = 0.006) while there was no convincing evidence of virus replication in VN04/H5N1 virus infected wd-NHBE cells **(** **Figure 5B** **)**. Furthermore, at 24 h post infection, the titers of virus in VN04/H5N1 infected ud-NHBE was significantly higher (*p* = 0.005) than in wd-NHBE.

Both viruses replicated productively in ud-NHBE cells but VN04/H5N1 had a significantly higher viral yield than HK98/H1N1 at 24 h post infection (*p* = 0.049) **(** **Figure 5C** **, grey bar)**. In contrast, influenza HK98/H1N1 replicated more efficiently than VN04/H5N1 in the wd-NHBE cells (*p* = 0.039) **(** **Figure 5C** **, black bar)**.

We also demonstrated that the low pathogenic HK98/H1N1 virus can replicate efficiently in both ud- and wd-NHBE cell cultures in the absence of exogenous trypsin. On the other hand, there was no convincing evidence of VN04/H5N1 virus replication in wd-NHBE cells with or without the addition of exogenous trypsin (data not shown).

### Cytokine and Chemokine mRNA Induction by Influenza Virus Infected NHBE Cells

**Figure 6:**
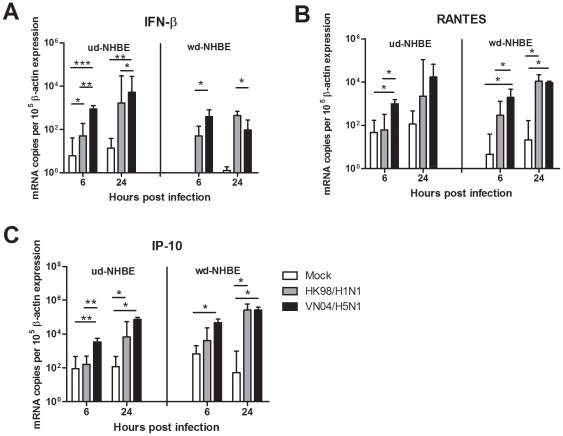
The (A) IFN-β, (B) RANTES and (C) IP-10 gene expression of the ud- and wd-NHBE cells at 6 h and 24 h post infection of HK98/H1N1 and VN04/H5N1. The chart showed the mean and the standard error from three independent experiments. Single asterisk indicated statistically significant difference of means with *p*<0.05, double asterisks indicated statistically significant differences of means with *p*<0.01 and triple asterisks indicated statistically significant differences of means with *p*<0.001.

We next investigated the influence of the differentiation state of NHBE cells on the cytokine and chemokine responses induced by HK98/H1N1 and VN04/H5N1 viruses. Specifically, we wanted to determine whether the difference in NHBE cell differentiation (ud-NHBE Vs wd-NHBE) led to qualitative or quantitative differences in the profile of cytokines induced. Cytokine (TNF-α, IFN-β) and chemokine (RANTES, IP-10) gene expression profiles were evaluated by qPCR. TNF-α mRNA was not expressed in either mock or both influenza A virus (HK98/H1N1 and VN04/H5N1) infected ud- and wd-NHBE cells (data not shown). In general, IFN-β, RANTES and IP-10 gene was induced following infection with influenza H1N1 and H5N1 virus in both the ud-NHBE and wd-NHBE cells. VN04/H5N1 virus led to significantly higher IFN-β **(** **Figure 6A, left panel** **)** and IP-10 **(** **Figure 6C, left panel** **)** mRNA expression in ud-NHBE cells compared with HK98/H1N1 or mock infection at both 6 h and 24 h post infection. VN04/H5N1 virus induced a significantly higher RANTES expression than HK98/H1N1 virus infected cells at 6 h post infection but not at 24 hours post infection **(** **Figure 6B** **)**.

In wd-NHBE cells, VN04/H5N1 induced significantly higher IFN-β **(** **Figure 6A, right panel** **)** and **RANTES (** **Figure 6B, right panel** **)** mRNA expression than HK98/H1N1 at 6 h post infection. However, at 24 h post infection, RANTES **(** **Figure 6B, left panel** **)** and IP-10 **(** **Figure 6C, right panel** **)** mRNA expression levels were comparable in VN04/H5N1 and HK98/H1N1 viruses infected while IFN-β mRNA expression level was significantly lower (*p* = 0.03) in VN04/H5N1 infected cells than in HK98/H1N1 infected cells **(** **Figure 6A, right panel** **)**. Inactivation of the virus by ultraviolet irradiation or high temperature (70°C for 15 minutes) prior to infection of the ud- and wd-NHBE cells abolished cytokine induction (data not shown). This suggests that virus replication was required for cytokine induction and also rules out the possibility that endotoxin contamination in virus stocks contributed to the observed cytokine responses. Besides, an increase in the MOI up to 5 did not result in changes in the expression profile of the cytokine and chemokine mRNA induced by the influenza H1N1 and H5N1 viruses in both the ud- and wd-NHBE cells (data not shown).

### Secretion of Cytokine and Chemokine Protein of Infected NHBE Cells

**Figure 7:**
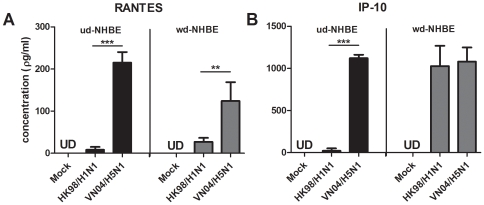
The (A) RANTES and (B) IP-10 protein of the supernatant collected at the apical compartment of the influenza HK98/H1N1 and VN04/H5N1 virus infected ud-NHBE (dark bars) and wd-NHBE (grey bars) cells at 24 h post infection. The chart showed the mean and the standard error from three independent experiments. Double asterisk indicated statistically significant difference of means with *p*<0.001 and triple asterisks indicated statistically significant differences of means with *p*<0.0001. UD indicated the protein concentration of the sample is below the detection limit.

We next used ELISA to measure the secretion of cytokine and chemokine proteins from ud- and wd-NHBE cell culture supernatants infected by influenza H1N1 and H5N1 viruses. The concentration of RANTES and IP-10 in the supernatants of mock infected cells was below the detection limit of the ELISA kit (31.25 ρg/ml and 62.5 ρg/ml, respectively). In parallel with the gene expression profile, at 24 h post infection, VN04/H5N1 led to significantly higher RANTES (*p*<0.001) **(Figure 7A, left panel)** **** and IP-10 (*p*<0.001) **(Figure 7B, left panel)** protein secretion in ud-NHBE cells compared with HK98/H1N1. While VN04/H5N1 induced significantly higher levels of RANTES secretion in wd-NHBE cells than did HK98/H1N1 (*p*<0.01) **(Figure 7A, right panel)**, the IP-10 protein secretion was comparable between in HK98/H1N1 and VN04/H5N1 infected cells. We failed to detect any IFN β protein in the supernatant of the NHBE cultures after influenza virus infection because of the poor sensitivity of the IFN-β ELISA (detection limit 250 ρg/ml).

## Discussion

The presence of ciliated and goblet cells [21] within the pseudostratified epithelium [22], the establishment of transepithelial resistance [20] and the expression of HAT protease in the bronchial epithelium are indicators of a well differentiated bronchial epithelium. The fact that a low-pathogenic H1N1 virus replicates efficiently in these cells without exogenous trypsin and that addition of trypsin did not further enhance viral replication suggests that the wd-NHBE cells make sufficient HAT to support low pathogenic influenza viral replication, as occurs *in vivo*. Taken together with the mucin producing ability, these findings provide good evidence that wd-NHBE cell system had acquired the physiological properties of a normal human bronchial epithelium and provides a relevant model for the study of influenza virus replication and pathogenesis.

We compared the distribution of Sia receptors in ud-NHBE and wd-NHBE cell models with that seen in human bronchial epithelium and found that the wd-NHBE has a comparable profile to human tissue while the ud-NHBE does not. These differences in Sia configurations and expression between ud-NHBE and wd-NHBE cells may explain at least in part, the different permissiveness to the human seasonal H1N1 and avian H5N1 viruses. The higher expression of Sia-α2-6 in wd-NHBE may account for the better replication of human influenza H1N1 virus which preferentially utilizes this receptor for entry. The lower abundance of Sia-α2-3Galβ1-3GalNAc in wd-NHBE may be associated with the lower permissiveness of these cells for avian influenza H5N1 virus (which has a higher binding affinity to Sia-α2-3Galβ1-3GalNAc). In addition to the degree of differentiation, the complexity of wd-NHBE (i.e. three-dimensional structure with a heterogeneous cell population vs. monolayer with single cell type) could also account for such differences.

Cell tropism of influenza A virus in wd-human bronchial epithelial cells was previously studied by Matrosovich *et al*.[23]. They found that human viruses preferentially infected non-ciliated cells while the avian viruses infected ciliated cells in the first cycle of infection. They correlated this observation with the localization of Sia receptors on these two cell types, non-ciliated cells express mainly Siaα2-6 receptors known to bind human influenza viruses while ciliated cells predominantly express Siaα2-3, the receptor for avian influenza viruses. Others have also reported that human influenza virus replicates more efficiently compared to avian influenza viruses in human differentiated tracheo-bronchial epithelial cells [24]. Our data are in agreement with these findings that the H5N1 virus replication in wd-NHBE cells was less efficient when compared to H1N1 virus. In contrast, as both Siaα2-3 and Siaα2-6 receptors were present in the ud-NHBE cells, with homogenous distribution in the majority of basal epithelial cells, both H1N1 and H5N1 viruses replicate well in these cells.

Mucin secreted by the goblet cells was present in wd-NHBE cell cultures. Extracts of human bronchial mucin are found to contain Sia primarily in the Sia-α2-3 linkage [25], [26] and much less Sia-α2-6 linkage [26]. Influenza virus inhaled to the conducting airway would first encounter this soluble form of Sia receptor in mucin before reaching the respiratory epithelial cell membrane with the Sia receptors that the virus needs to attach to in order to initiate infection. Secreted mucin with the Sia-α2-3 receptor has been postulated to act as binding-decoy for influenza H5N1 virus, reducing the possibility of the virus to bind to the bronchial epithelium and achieve productive replication in the airway. Matrosovich *et al* have noted that even after extensive washing of wd-human airway epithelial cell cultures, there is still lectin binding to the cell surface-associated mucins [23]. In addition, the continuous secretion of mucin by goblets cells in the wd-NHBE can interfere with multiple round of infection.

Bronchial epithelial cells play an important role in innate defense and in the pathogenesis of the influenza virus infection. They are target cells for infection and they secrete cytokines and chemokines upon infection which mediate host defense and potentially contribute to the inflammation of the bronchial mucosa. Influenza A virus (H3N2) infection of undifferentiated human bronchial epithelial cells NCI-H292 leads to induction of RANTES, IL-6 and IL-8 secretions, but not granulocyte-macrophage colony stimulating factor; these may be relevant in host defense and pathogenesis [27], [28]. There are however limited studies of cytokine responses in well differentiated bronchial epithelium. In our study of bronchial epithelial cells, influenza virus infection induced IFN-β, RANTES and IP-10 mRNA in these cells. Interestingly, a strikingly different cytokine and chemokine expression was observed in the ud-NHBE and wd-NHBE upon infection by the same virus. There were also differences between H1N1 and H5N1 influenza viruses in the profile of cytokine and chemokine release in ud-NHBE compared with wd-NHBE. At 6 h post infection of ud-NHBE, VN04/H5N1 virus led to a more potent expression of IFN-β, RANTES and IP-10 compared with HK98/H1N1 virus. This pattern is similar to the human peripheral blood derived macrophages [6] and pneumocytes [5].

On the contrary, at 24 h post infection, wd-NHBE cells had an apparently reverse IFN-β mRNA profile with VN/04/H5N1 virus inducing significantly lower levels than HK98/H1N1. Moreover, VN04/H5N1 virus did not significantly increase expression of the chemokine genes RANTES and IP-10 compared with HK98/H1N1 (Figure 6). These may be associated with the lower levels of H5N1 viral replication in wd-NHBE cells as evidenced by M gene levels (Figure 3), imunofluorescent cells (Figure 4) and non-productive replication (Figure 5B).

Zeng *et al*. [12] previously reported that IFN-β expression in Calu-3 cells at 24 h post infection with H5N1 was lower and more delayed than seen with seasonal influenza (H3N2). They found that this delayed IFN-β response was associated with delayed induction of interferon regulatory factor-3 nuclear translocation and suggested that H5N1 evades the IFN-β innate host defenses allowing it to replicate more efficiently. Our findings in NHBE cells are in agreement with theirs at 24 h post infection but we find this is associated with lower (not higher) levels of virus replication. Furthermore we find that at 6 h post infection, H5N1 virus induces more IFN-β than H1N1 virus. These apparently contradictory findings may partly reflect the cell type (Calu-3 vs. NHBE) and the extent of cell differentiation in the two experimental models and the time of data collection. Calu-3 is a transformed continuous cell line originally isolated from a human pulmonary adenocarcinoma and they were differentiated in transwell culture inserts for one week only. In contrast, our wd-NHBE cells are primary cells derived from normal human bronchi, and are cultured and differentiated for 21 days in ALI. As shown in figure 1A, NHBE cell differentiation in our hands was not completed after seven days of ALI cultures when compared to day 21 (Figure 1B). Interestingly, studies on the pathogenesis of severe acute respiratory syndrome coronavirus (SARS-CoV) using polarized Calu-3 cells and differentiated human airway epithelial cells [29], [30] found that the latter supported a more productive replication of SARs-CoV. Since SARS-CoV infects ciliated cells, this difference was attributed to the lower numbers of ciliated epithelial cells obtained in the polarized Calu-3 cell model [31]. It is possible that the wd-NHBE cell model which recapitulates the morphological and physiological features of the human conducting airway *in vivo* would be a more representative model to study respiratory infection.

In conclusion, our data shows that the origin and differentiation of bronchial epithelial cells has a major impact to the permissiveness of cells to influenza A virus replication and on the host responses elicited by such infection. This reinforces the importance of the use of physiologically relevant models for the understanding of influenza pathogenesis. It is relevant to note that a regenerating epithelium recovering from mucosal damage caused by viral or other agents would have the equivalent of undifferentiated epithelial cells present, thus the data from ud-NHBE may still be physiologically relevant to such a situation.
